# Highly Selective Biomimetic Flexible Tactile Sensor for Neuroprosthetics

**DOI:** 10.34133/2020/8910692

**Published:** 2020-08-24

**Authors:** Yue Li, Zhiguang Cao, Tie Li, Fuqin Sun, Yuanyuan Bai, Qifeng Lu, Shuqi Wang, Xianqing Yang, Manzhao Hao, Ning Lan, Ting Zhang

**Affiliations:** ^1^i-Lab, Key Laboratory of Multifunctional Nanomaterials and Smart Systems, Suzhou Institute of Nano-Tech and Nano-Bionics (SINANO), Chinese Academy of Sciences (CAS), 398 Ruoshui Road, Suzhou 215123, China; ^2^School of Nano-Tech and Nano-Bionics, University of Science and Technology of China, 96 Jinzhai Road, Hefei, Anhui 230026, China; ^3^Laboratory of Neurorehabilitation Engineering, School of Biomedical Engineering and Institute of Medical Robotics, Shanghai Jiao Tong University, 1954 Huashan Road, Shanghai 20030, China; ^4^Center for Excellence in Brain Science and Intelligence Technology, Chinese Academy of Sciences, Shanghai 200031, China

## Abstract

Biomimetic flexible tactile sensors endow prosthetics with the ability to manipulate objects, similar to human hands. However, it is still a great challenge to selectively respond to static and sliding friction forces, which is crucial tactile information relevant to the perception of weight and slippage during grasps. Here, inspired by the structure of fingerprints and the selective response of Ruffini endings to friction forces, we developed a biomimetic flexible capacitive sensor to selectively detect static and sliding friction forces. The sensor is designed as a novel plane-parallel capacitor, in which silver nanowire–3D polydimethylsiloxane (PDMS) electrodes are placed in a spiral configuration and set perpendicular to the substrate. Silver nanowires are uniformly distributed on the surfaces of 3D polydimethylsiloxane microcolumns, and silicon rubber (Ecoflex®) acts as the dielectric material. The capacitance of the sensor remains nearly constant under different applied normal forces but increases with the static friction force and decreases when sliding occurs. Furthermore, aiming at the slippage perception of neuroprosthetics, a custom-designed signal encoding circuit was designed to transform the capacitance signal into a bionic pulsed signal modulated by the applied sliding friction force. Test results demonstrate the great potential of the novel biomimetic flexible sensors with directional and dynamic sensitivity of haptic force for smart neuroprosthetics.

## 1. Introduction

**Figure 1:**
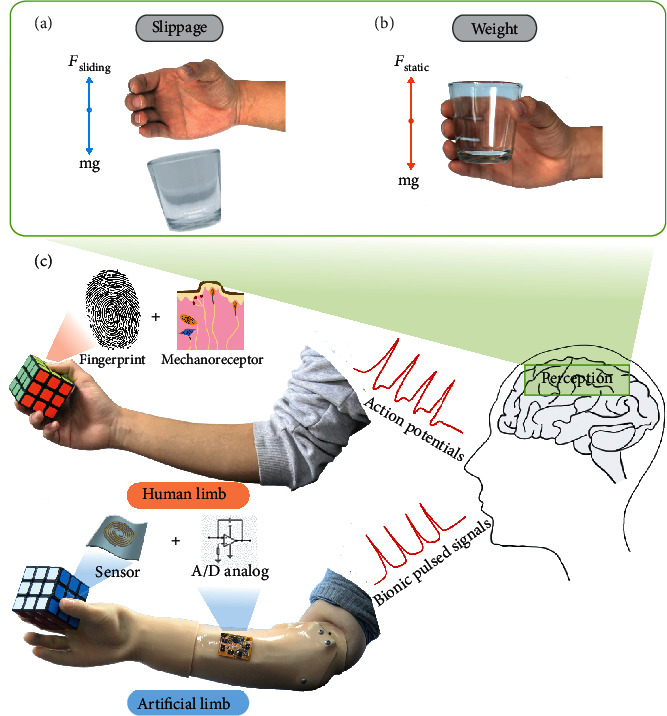
Schematic illustration of the biomimetic flexible friction force sensor for dexterous neuroprosthetics. (a, b) The significance of static and sliding friction forces in daily life. The perception of slippage allows people to grasp the target objects more stably by adjusting the gripping strength without visual aid. The perception of the static friction force is closely related to estimating the weight of an object. With the perception of slippage and weight, prosthetics become “smarter” and are able to achieve some complex tasks without visual aid. (c) The bionic mechanism of neuroprosthetics to regenerate the perception of touch. For human touch, two key factors are fingerprints and four types of mechanoreceptors, which selectively respond to different types of forces. We designed a fingerprint-structured flexible capacitive sensor with custom-designed signal encoding circuit to mimic Ruffini ending functions. The output bionic pulsed signals can be transferred to the nerve tissue.

Recently, flexible bionic sensors have attracted notable research interest and have been envisioned as key technologies for the applications of neuroprosthetics [1–4], robotics [5–8], and human-machine interactions [9–11]. Especially for neuroprosthetic systems, flexible sensors assembled on prosthetic hands provide front-end sensory signals for subsequent signal encoding, transmission, and neural interfacing, resulting in the regeneration of bionic tactile information [5, 12]. However, the abandonment rate of artificial limbs is high, and the applications of robotic hands are still not popular at present. One of the reasons is that these hands are not dexterous enough to manipulate objects in practice and are limited by unknown information such as slippage and weight perception. Sliding friction force is a critical criterion governing whether hands can grab objects stably (Figure 1(a)), and static friction force is crucial for people to estimate the weight of an object in hand (Figure 1(b)). Therefore, to improve the intelligent and manipulative levels of prosthetic and robotic hands, the detection of static and sliding friction forces is necessary for dexterous in-hand manipulation.

In the past several years, there have been many studies on flexible force sensors with great performance [13, 14], some of which have already surpassed the sensitivities of human beings [15, 16]. However, the detection of sliding and static friction forces has been overlooked. Most of the reported flexible force sensors are sensitive to yet not selective of multitype force stimuli. To address the requirement of a selective response to multitype forces, until recently, a few technologies, such as capacitive sensor arrays [5] and triboelectric nanogenerator (TENG) arrays [17], have been designed to detect the shear force. Clementine et al. proposed capacitive sensor arrays based on the 3D hill structure, with each hill corresponding to 25 capacitor pixels on top of and around the hill. Due to the anisotropic deformation of the 25 sensor pixels under multidirectional pressure, the sensor array can measure and discriminate both normal and shear forces. With the following control program for robot arms, capacitive sensor arrays provide sensing feedback for controlling a robot arm in various tasks [5]. Ren et al. designed a triboelectric nanogenerator array with a four-partitioned electrode structure. The four electrodes of the sensors react nonuniformly under a shear force, resulting in the ability to detect both normal and shear forces. However, these sensor arrays still cannot distinguish between the specific static friction force and sliding friction force. In addition, the formation of the as-assembled array also increases the complexity of signal processing for practical applications. A specific sensor that can detect and differentiate the normal force, static friction force, and sliding friction force, to the best of our knowledge, has not been developed.

To regenerate the bionic tactile perception of “smart” neuroprosthetics, a sensor design can learn from the tactile perception mechanism of fingers (Figure 1(c)). Fingers have superior sensitivity and multiple tactile sensation abilities compared with other parts of the body, benefitting from the combined effects of fingerprint epidermal morphology and four types of mechanoreceptors interred in the dermis [18]. The morphology of fingerprints is an uneven spiral, which is attributed to the perception of texture and sensitivity [19]. Four types of mechanoreceptors (Meissner corpuscles, Merkel cells, Ruffini endings, and Pacinian corpuscles) are able to efficiently convert various mechanical stimuli into physiological spike signals (Figure 1(c)), and then, the action potential signals representing information are transmitted to the somatosensory cortex by the nerve bundles in submilliseconds [20]. Mechanoreceptors distributed in different regions of the skin on the hand have selective sensitivity to different types of forces. Particularly, Ruffini endings are located in the dermis, which has directional preferences [21–26]. Benefiting from the best response ability to skin stretch [22], Ruffini endings are sensitive to the shear forces containing the static and sliding friction forces during object manipulation [23]. The larger the shear force is, the higher the frequency of response spike signal will be [26].

Inspired by the responsive function of Ruffini endings to friction forces, we design and fabricate unique fingerprint-like flexible capacitive sensors with selective sensitivity to nonnormal forces (static and sliding friction forces). The capacitance of the sensor remains constant when normal force is applied, increases when static friction force is applied, and decreases when sliding occurs. Furthermore, we demonstrate that the specific response of the sensors can be used for sliding detection and object weight recognition in robotic hands. In addition, the circuit for encoding the biomimetic output is custom-designed to resolve the signal incompatibility between the flexible sensors and the nervous system, which is useful in the transfer of sensing signals from neuroprosthetics to amputees via appropriate neural interfaces [27, 28].

## 2. Results

### 2.1. Design and Characterization of Flexible Friction Force Sensors

Typical capacitance sensors are usually designed with a plane-parallel capacitor structure, containing an intermediate dielectric layer and two electrode plates: one on the top and one on the bottom. The classic equation (1) of the plane-parallel capacitor is as follows:

(1) $$\begin{array}{c} C=\frac{\varepsilon_{r}\varepsilon_{0}S}{d}, \end{array}$$

where *C* is the capacitance, *ε*_r_ is the relative permittivity, *ε*_0_ is the permittivity of free space, *S* is the effective overlapping area between the two capacitance plates, and *d* is the vertical distance between the two plates. According to this structure design, for flexible capacitive sensors, flexible thin film electrodes at the top will deform elastically when an external force is applied, which will lead to a decreased vertical distance (*d*) and increased capacitance regardless whether the direction of the force is perpendicular or parallel to the sensor. Thus, it is difficult for these traditional flexible capacitive sensors to discriminate the different types of force.

**Figure 2:**
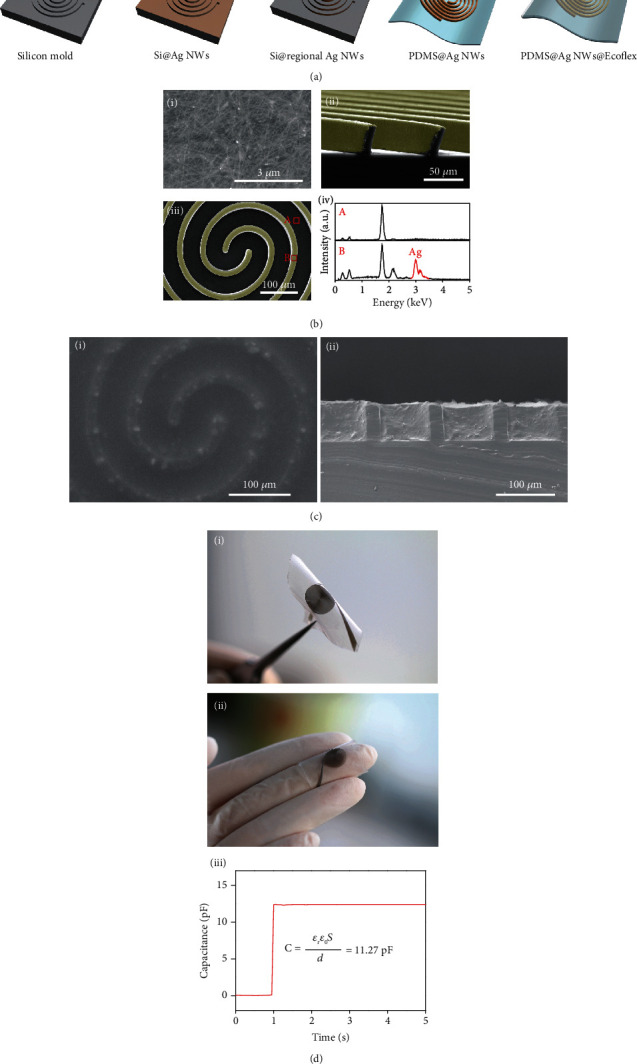
Fabrication and characterization of flexible shear force sensors. (a) A schematic illustration of the sensor fabrication process. (b) Scanning electron microscopy (SEM) images of the PDMS@Ag NWs. (i) SEM image of Ag NWs on PDMS. (ii) Side-view SEM image showing that Ag NWs were uniformly distributed on the top and sidewalls of the spiral microcolumns. (iii) Top-view SEM image. (iv) Energy spectra of the spiral column and the substrate. No Ag NWs existed between the microcolumns. (c) SEM images of PDMS@Ag NWs@Ecoflex. (i) Top-view SEM image. (ii) Side-view SEM image showing that Ecoflex filled the air gap evenly. (d) (i) Photograph showing the flexibility of the sensor. (ii) Photograph of a fabricated sensor. (iii) The actually measured capacitance of the fabricated sensor, which is in accordance with the theoretical capacitance of approximately 11.27 pF.

Inspired by the morphology of the fingerprint, we propose a novel flexible capacitor, the capacitance plates of which are spiral and perpendicular to the substrate. The spiral is centrosymmetric, which ensures the similar sensitivity to shear force from any direction in plane (Figure S5). The sensor consists of silver nanowire (Ag NW)–3D polydimethylsiloxane (PDMS) electrodes and silicon rubber (Ecoflex®) dielectrics. The fabrication process is shown in Figure 2(a). To balance the capacitance and size of the sensors, spiral electrodes with different height-width ratios were designed with a fixed width (15 *μ*m), a fixed spacing (50 *μ*m) between two adjacent electrodes, and different heights of 15 *μ*m, 25 *μ*m, and 35 *μ*m. First, a silicon wafer mold with spiral grooves was fabricated by plasma etching. Second, Ag nanowires (30 nm diameter, 20 *μ*m length) were spray-coated onto the silicon wafer mold. Then, the Ag nanowires on the top layer of the Si substrate were removed by scraping with the inclined plane of a syringe, and subsequently, the PDMS mixture was coated onto the substrate and peeled off after being completely cured for 3 hours at 80°C. Through the replication process, Ag nanowires were embedded into the surface of the 3D PDMS microcolumn (Figure 2(b) i). Figure 2(b) ii is a crossview scanning electron microscopy (SEM) image of PDMS demolded from the silicon mold, showing column-type electrodes and Ag nanowires that are highlighted in green. In particular, the top-view SEM image (Figure 2(b) iii) with the analysis of the energy dispersive system (EDS) (Figure 2(b) iv) demonstrates that no Ag nanowires existed between the microcolumns, which ensured insulation between the two electrodes. Finally, silicon rubber (Ecoflex®) was employed as the dielectric to fill the grooves between the microcolumns (Figure 2(c)). The Ecoflex-Ag NW-PDMS sandwich structure prevents the shedding and oxidation of Ag nanowires. Microcolumn electrodes could still be easily deformed under shear force due to the lower Young modulus (0.13 MPa) of Ecoflex [29] compared to that of PDMS (3 MPa) [30]. The resulting sensors were flexible (Figure 2(d) i) and had the proper size (2 cm × 2 cm) for prosthetic applications (Figure 2(d) ii). According to Equation (1), the theoretical capacitance was approximately 11.27 pF, which is in accordance with the measured capacitance (12.41 pF) of the as-assembled bionic sensor, proving the validity of this capacitive microstructure design (Figure 2(d) iii).

### 2.2. Response and Sensing Mechanism of the Flexible Friction Force Sensors

**Figure 3:**
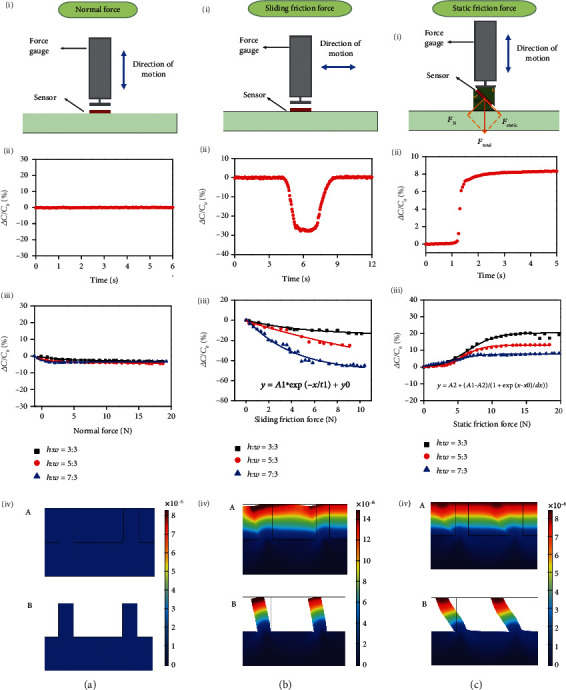
Response ability of the flexible shear force sensors and its corresponding mechanism. (a) Normal force: (i) experimental setup. (ii) The response curve for an 11.2 N (199.1 kPa) normal force. The capacitance was kept basically constant. (iii) The different height-width ratio responses of the sensor to the 0-20 N (0-355 kPa) normal force. (iv) Finite element analysis (FEA) showing that the structure remains stable under 14 kPa of pressure. (A) The deformation of the film containing electrodes and dielectric. (B) The deformation of electrodes specifically. (b) Sliding friction force: (i) experimental setup, in which the force gauge moves parallel to the sensor. (ii) The response curve for a 3.8 N (67.6 kPa) sliding friction force. The capacitance decreased initially and returned to its original value when the force gauge left the sensor. (iii) The different height-width ratio responses of the sensor to the 0-10 N (0-178 kPa) sliding friction force. The capacitance variations follow the fitting formula Δ*C*/*C*_0_ = *A*∗*e*^−*F*_sliding_/*B*^ + *C*. (iv) Finite element analysis showing that the electrodes were laterally bent when a 7 kPa sliding friction force and 14 kPa pressure were applied. (A) The deformation of the film containing electrodes and dielectric. (B) The deformation of electrodes specifically. (c) Static friction force: (i) experimental setup, in which the force gauge moves vertically and the sensor is fixed on the oblique plane of 45°. (ii) The response curve for an 11.2 N (199.1 kPa) static friction force. The capacitance increased immediately. (iii) The different height-width ratio responses of the sensor to the 0-20 N (0-355 kPa) static friction force. The capacitance variations follow the fitting formula Δ*C*/*C*_0_ = *a* − *b*/(1 + *e*^(*F*_static_ − *c*)*F*_static_−*c*/*d*^). (iv) Finite element analysis (FEA) showing that the electrodes were laterally stretched when a 14 kPa static friction force and 14 kPa pressure were applied. (A) The deformation of the film containing electrodes and dielectric. (B) The deformation of electrodes specifically.

To investigate the capacitive response to the applied normal force, static friction force, and sliding friction force, the testing apparatuses were set up, consisting of a force gauge and a computer-controlled moving stage (Figures 3(a) i–3(c) i). The motion direction of the force gauge has two degrees of freedom: parallel and perpendicular relative to the tested sensors. For the normal force, pressure was applied to the sensor through the vertical movement of the force gauge (Figure 3(a) i). For the sliding friction force (Figure 3(b) i), sliding was applied through the horizontal movement of the force gauge at a constant speed. The force gauge probe gradually contacts the sensor from one side and leaves it from the other side. According to the classic friction law,

(2) $$\begin{array}{c} F_{\text{sliding}}=\mu F_{\text{N}}, \end{array}$$

where *F*_sliding_ is the sliding friction force, *μ* is the coefficient of sliding friction, and *F*_N_ is the normal force. In this research, *μ* was fixed at 0.65 [31]. As *F*_N_ changes, *F*_sliding_ changes.

For the static friction force, the sensor was fixed on an oblique plane of 45° (Figure 3(c) i). Therefore, the normal force and the static friction force applied to the sensor were equal during the vertical movement of the force gauge. According to the force analysis in

(3) $$\begin{array}{c} F_{\text{total}}={\sqrt{2}}^{*}F_{\text{static}}, \end{array}$$

*F*_total_ is the value displayed on the force gauge, and *F*_static_ is the applied static friction force.

The sensitivity of a capacitive sensor is defined as

(4) $$\begin{array}{c} \frac{\Delta C}{C_{0}}=\frac{C-C_{0}}{C_{0}}, \end{array}$$

where *C* and *C*_0_ are the measured capacitance and the initial capacitance before applying force, respectively. Figures 3(a) ii–3(c) ii show the real-time response curves under the normal, static friction, and sliding friction forces (height : width = 7 : 3), respectively. As shown in Figure 3(a) ii, the capacitance remained nearly constant when an 11.2 N (119.1 kPa) normal force was applied to the sensor. During the sliding of the force gauge across the sensor (*F*_sliding_ = 3.8 N (67.6 kPa)), the capacitance decreased initially and then returned to its original value when the force gauge left the sensor (Figure 3(b) ii). In contrast, when an 11.2 N (119.1 kPa) static friction force was applied, the capacitance increased immediately (Figure 3(c) ii). These results reveal that the type of force can be readily distinguished according to the capacitive signal change due to the selective response.

Moreover, the height-width ratio is a key parameter of the sensitivity. Structures were designed with the same electrode distance and three different aspect ratios of 3 : 3, 5 : 3, and 7 : 3. As shown in Figure 3(a) iii, the capacitance remained nearly constant when applying the normal force (0-20 N) (0-355 kPa) regardless of the change in the height-width ratio. The capacitance decreased with increasing sliding friction force (Figure 3(b) iii) following the fitting formula Δ*C*/*C*_0_ = *A*∗*e*^−*F*_sliding_/*B*^ + *C*. *A*, *B*, and *C* are constant terms, as shown in Table 1. This demonstrates that the higher the height-width ratio is, the higher the sensitivity of the sensors to the sliding friction force (Figure 3(b) iii). In contrast, the capacitance increased with increasing static friction force (Figure 3(c) iii). The capacitance variations followed the fitting formula Δ*C*/*C*_0_ = *a* − *b*/(1 + *e*^(*F*_static_ − *c*)*F*_static_−*c*/*d*^). *a*, *b*, *c*, and *d* are constant terms, as shown in Table 1. The lower the height-width ratio is, the higher the sensitivity of the sensors to the static friction force (Figure 3(c) iii).

To elucidate the underlying mechanism, deformations of the electrodes were investigated by performing finite element analysis (FEA). Different from interior voids (i.e., a foam structure), the bulk structure with the Ecoflex-filled microgrooves exhibited low structural compressibility, resulting in insensitivity to the normal force, as exemplified by the FEA results in Figure S1. Figures 3(a) iv–3(c) iv show the deformations of the electrodes via FEA when the normal force, sliding friction force, and static friction force were applied. The forces applied through the top silica glass and the bottom of the sensors were fixed during the analysis. When only the normal force (11.4 Pa) was applied, no obvious deformation in the structure of the electrodes was observed (Figure 3(a) iv), leading to a constant capacitance. Under the sliding friction force (7 kPa) and normal force (14 kPa), the electrodes are laterally bent but not stretched (Figure 3(b) iv). Although *d* remains unchanged, a decrease in *S* caused by the lateral bending of the electrodes leads to the decreased capacitance during sliding. Conversely, when the static friction force (14 kPa) and normal force (14 kPa) are applied, there is no relative displacement at the contact surface, and the electrodes are laterally stretched (Figure 3(c) iv). The increased capacitance is caused by the increased *S* and the decreased *d*. The detailed analyzation of changes in *S* and *d* is listed in supplementary materials (Figure S2).

### 2.3. Slippage Detection and Weight Perception of Flexible Friction Force Sensors

**Figure 4:**
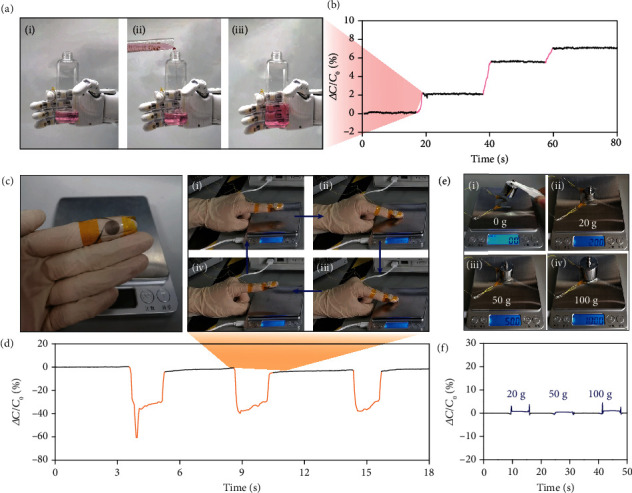
Demonstrations of the Ruffini-ending-inspired flexible shear force sensor with the ability to selectively respond to static and sliding friction forces. (a, b) The perception of weight related to the static friction force. The robotic hand equipped with the sensor grasped a bottle with a constant force. The capacitance increased as the water volume increased, which corresponded to the static friction force. (c, d) The perception of slippage related to the sliding friction force. A glove equipped with the sensor was put on to perform the sliding action. The capacitance decreased immediately as soon as sliding began. (e, f) The characteristic nonsensitivity to the normal force. Weights of 20 g, 50 g, and 100 g were put on this Ruffini-ending-inspired sensor, in that order.

Benefiting from the ability of flexible biomimetic sensors to selectively respond to static and sliding friction forces, we set up the corresponding prosthetic demonstration scenarios (Figure 4). First, to demonstrate the ability to detect the weight, a robotic hand mounted with a Ruffini-ending-inspired sensor was set to grasp a plastic bottle with a constant force (Figure 4(a)). Then, water was gradually poured into the bottle by a graduated cylinder to increase the static friction force. The static friction force was the only variable in this process. As shown in Figure 4(b), the capacitance of the sensor increased as the 12 mL of water was poured in real time (movie S1), and the capacitance remained at the steady state after the pouring of water was stopped. This action was repeated three times to demonstrate the ability of weight perception.

Then, the slippage detection ability was demonstrated by using a glove equipped with a Ruffini-ending-inspired sensor, which was put on the hand to perform the sliding action (Figure 4(c)). During sliding, the friction force was exerted on the sensor. The sliding action was repeated three times. As movie S2 shows, during the sliding process, the normal force immediately increased to 0.5 N in the early stage and then decreased gradually. The corresponding capacitance substantially decreased by 40% once slippage occurred and then returned to the original value immediately when the slippage stopped (Figure 4(d)). These results demonstrate the slippage detection ability of flexible friction force sensors.

Finally, the insensitivity to the normal force is demonstrated in Figure 4(e) and movie S3, where standard weights of 20 g, 50 g, and 100 g were put on the Ruffini-ending-inspired sensor, in that order. The corresponding capacitive responses are shown in Figure 4(f). When the weights were put down or taken away, two spikes were generated due to the static friction force caused by the nonvertical motion. Overall, the capacitance that changes under these different weights were less than 1%, showing that the sensor is insensitive to the normal force.

### 2.4. The Bionic Behavior of Flexible Friction Force Sensors

**Figure 5:**
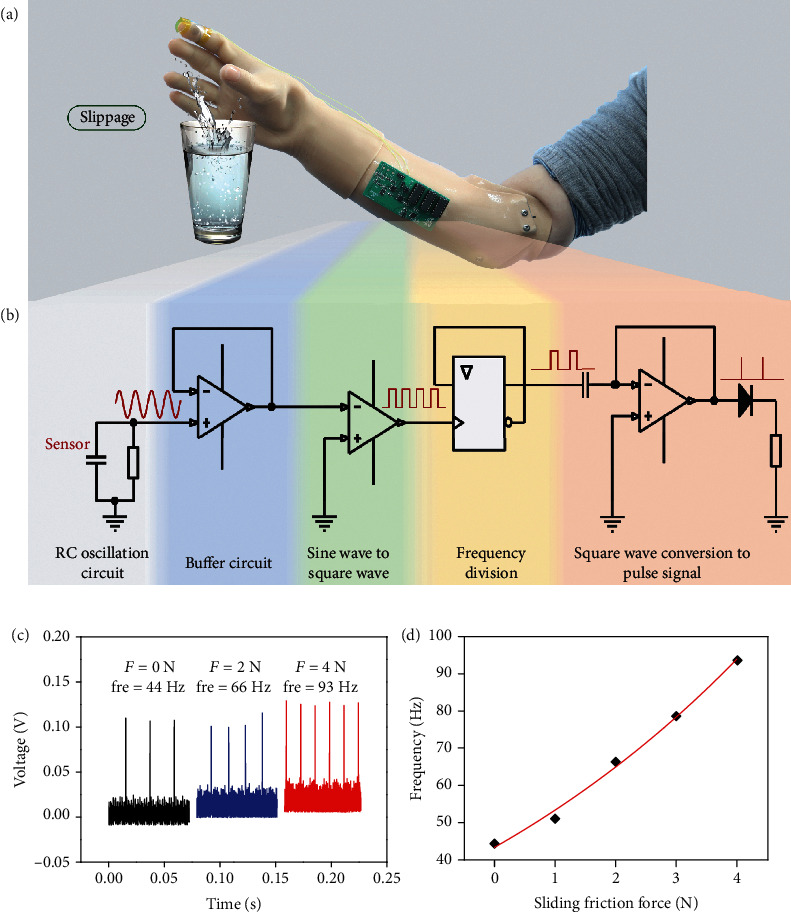
The bionic signal encoding of the Ruffini-ending-inspired flexible sensor. (a) Illustration of prosthesis sliding perception. The Ruffini-ending-inspired sensor with processing circuits is applied to a prosthetic hand of a woman with a lower-arm amputation. (b) Schematic diagram showing a circuit that converts analog signals recorded from the sensor into low-frequency pulse signals (nerve-like signals). (c) The pulse shape through the processing circuit. The pulse frequency changed with the applied sliding friction forces. (d) The pulse frequency responses in the 0 to 4 N range of the sliding friction force.

The ability to distinguish slippage is still lacking for neuroprosthetics, limiting dexterous manipulation. With the perception of slippage, humans can naturally estimate whether an object is grasped without visual aids, which is meaningful for daily life. The expression of tactile information is an action potential based on biologically driven models in the central nervous system, the frequency of which conveys tactile sensation to the somatosensory cortex. In addition, the frequency of spikes increases with increasing applied force [26, 32, 33]. However, for neuroprosthetics, slippage detection at the sensor level is not sufficient due to the absence of proprioception. To regenerate the slippage perception of neuroprosthetics (Figure 5(a)), a signal encoding circuit was designed for bionic stimulus response signals, which is hopeful to transmit analog signals from the sensors into the nerve tissue.

To achieve the above expected functions, a signal encoding circuit was assembled specifically for this Ruffini-ending-inspired sensor, as shown in Figure 5(b). First, the recorded capacitance signal was transformed into a sine signal through a Wien bridge oscillation circuit, which can be easily adjusted within a wide frequency range. The oscillating circuit is composed of a capacitance sensor (*C*), a matching capacitor (*C*_1_), and two matching resistors (*R*_1_ and *R*_2_), forming an RC series-parallel network. When the positive and negative feedback of the operational amplifier circuit is in equilibrium, the oscillation can continue. At this time, the output waveform frequency follows formula

(5) $$\begin{array}{c} f=\frac{1}{2\sqrt{R_{1}R_{2}CC_{1}}}, \end{array}$$

where *f* is the frequency of the sine signal and *R*_1_ and *R*_2_ are the matching resistances with known resistance values (*R*_1_ = *R*_2_ = *R*). *C*_1_ is the matching capacitor with a fixed capacitance, and *C* is the capacitance of the sensor. Therefore, the relation between the oscillation frequency and the capacitance of the sensor can be simplified as shown in

(6) $$\begin{array}{c} f=\frac{1}{a\sqrt{C}}, \end{array}$$

where *a* is a constant.

Next, the high-frequency sine signal was transformed into the same frequency square wave through buffer and signal conversion circuits. To fit the vibration frequency range of mechanoreceptors (0~400 Hz), the square wave was processed by frequency division [20]. Last but not least, the divided squared wave was transformed into a bionic spike, which makes it possible to connect the sensors to the nervous system.

According to the signal encoding circuit, the frequency of the resulting bionic pulsed signal was modulated by the applied sliding friction force. Figure 5(c) illustrates the signal responses under sliding friction loadings of 0, 2, and 4 N. The spike frequency increased as the capacitance decreased due to slippage. As shown in Figure 5(d), the fitting formula of *f* = 63.21*e*^*x*/6.79^ − 20.02 was obtained with loading in the range of 0 to ~4 N. Moreover, a glove equipped with the designed sensor and the circuit was put on a hand to perform the sliding action (movie S4), which proved the feasibility of the bionic signal encoding circuit. The designed Wien bridge oscillator circuit is jam-proof and easy to realize and can be easily adjusted within a wide frequency range. Through the designed signal encoding circuit, the frequency range and variation trend of the sensors are able to achieve similar results to the human response to force stimuli and thus could endow neuroprosthetics with the ability of sliding perception.

## 3. Conclusion

In this work, we developed a Ruffini-ending-inspired flexible sensor with friction force selectivity. The sensor can not only measure shear forces with no response to the normal force but also further discriminate the static friction force and sliding friction force according to the variation tendency of the capacitance. Through FEA simulations, the mechanism of the selective response capacitor was concluded to be the deformation models of the vertical double helix architecture of the capacitance plates. Furthermore, we designed conversion circuits to code the sensor's analog signals into bionic stimulus signals, which may be transmitted to either the central nervous system (CNS, the brain and spinal cord) or the peripheral nervous system (PNS, the muscle or peripheral nerve electrical activity). The novel biomimetic flexible sensor presented in this work is meaningful and important not only for neuroprosthetics but also for human-machine fusion, such as wearable robots and exosuits.

## 4. Materials and Methods

### 4.1. Device Fabrication

#### 4.1.1. Fabrication of the Silicon Wafer with Fingerprint-like Microgrooves

Silicon molds with spiral microgrooves of different depth-width ratios were fabricated by traditional lithography and dry etching processes.

#### 4.1.2. Fabrication of Flexible Friction Force Sensors

A replication method was employed to prepare a spiral-column-type capacitive sensor. (i) Ag nanowires (30 nm diameter, 20 *μ*m length) were dispersed in ethanol, and the Ag nanowire concentration was 1 mg/mL. The silicon mold with microgrooves was soaked in trimethylchlorosilane for approximately 30 minutes, which was used as the mold-release agent. (ii) Then, silver-coated copper wires as electrodes were fixed onto the groove end of the silicon mold. Ag nanowire solution (2 mL) was sprayed onto the silicon mold and electrodes. Next, to form two plates of a capacitor, Ag nanowires at the sidewalls and bottom of the microgrooves were retained, but Ag nanowires on the top layer of the silicon wafer were scraped off. (iii) The PDMS prepolymer and its curing agent (Sylgard-184, Dow Corning) were stirred for 20 minutes with a ratio of 7 : 1 (*w*/*w*). Poured PDMS was cured at 80°C for 3 hours. With the aid of a mold-release agent, the Ag NW-PDMS composite thin film was easily peeled off from the silicon wafer without any damage. (iv) Last, because Ag NWs fall off easily during large deformation, Ecoflex with a low Young modulus was chosen to fill in the gaps. Part A and part B of the platinum cure silicone rubber compound were diluted in n-hexane at a ratio of 1 : 1 : 4 by weight. After stirring the diluted Ecoflex, the dispersion liquid was spin-coated onto the Ag NW-PDMS composite thin film at different revolutions per minute according to the height of the microcolumns. After spin-coating, the sensor was cured in a vacuum oven at 70°C for 30 minutes.

#### 4.1.3. Device Characterization

Capacitance measurements were taken using the Agilent B1500A semiconductor device analyzer. Capacitances were measured at a 1 MHz frequency with a 250 mV AC signal. Three kinds of forces were applied by a customized apparatus, which contains a *z*-axis electric moving stage with a force gauge and an *x*-axis electric moving stage (Beijing Optical Century Instrument Co., Ltd., SC100 series stepper motor controllers). In addition, the bionic spikes that occurred through the conversion circuits were recorded by an oscilloscope (Tektronix DPO5034B). DC voltage was provided by the voltage meter (RIGOL, DP832).

## Figures and Tables

**Table 1:** The fitting formula for the sensitivities to the sliding and static friction forces.

Height-width ratio	$\Delta C/C_{0}=A*e^{\frac{-F_{\text{sliding}}}{B}}+C$			$\Delta C/C_{0}=a-\frac{b}{1+e^{\left(F_{\text{static}}-c\right)/d}}$			
	*A*	*B*	*C*	*a*	*b*	*c*	*d*
3 : 3	15.0	5.31	-14.87	20.48	21.76	6.36	2.12
5 : 3	73.9	21.09	-74.71	13.05	13.44	5.77	1.45
7 : 3	52.9	4.08	-51.15	7.89	8.56	3.89	2.10

## Supplementary Materials

Supplementary 1 Figure S1: deformation under 200 MPa of normal force observed by finite element analysis. Figure S2: analysis of changes in *S* and *d* under static (A) or sliding friction forces (B). Figure S3: changing process of the capacitance that the static converted to sliding friction force. Figure S4: the pulse-like signal under static friction force. (A) The pulse frequency responses in the 0 to 8.5 N range of static friction force. (B) The pulse shape through the custom-designed circuit. The pulse frequency decreased with the applied static friction force. Figure S5: comparison between traditional parallel structure and spiral structure. The spiral is centrosymmetric, which ensures the same sensitivity to shear force from any direction in plane. Table S1: the method for distinguishing shear forces.

Supplementary 2 Movie S1: weight perception of the flexible friction force sensors. A robotic hand was set to grasp a plastic bottle with a constant force. The capacitance of the sensor increased with the addition of water.

Supplementary 3 Movie S2: slippage detection of the flexible friction force sensors. The flexible friction force sensor was mounted onto a wearable glove. The capacitance of the sensor decreased once slippage occurred.

Supplementary 4 Movie S3: insensitivity to the normal force of the flexible friction force sensors. The capacitance of the sensor was constant under different standard weights placed on the sensor.

Supplementary 5 Movie S4: the bionic behavior of flexible friction force sensors. The flexible friction force sensor mounted onto a wearable glove. The glove was used to apply a sliding friction force. The output is the frequency output through the conversion circuits. The frequency increased once slippage occurred.

Supplementary 6 Movie S5: changing of the capacitance as the static friction force converted to sliding friction force. A glove equipped with a Ruffini-ending-inspired sensor was put on hand to perform the action. The sensor was exerting static friction force at the beginning and then converted to slide mode.
