# Supplementary material for: Highly Selective Biomimetic Flexible Tactile Sensor for Neuroprosthetics
Source: Research (Wash D C). 2020 Aug 24;2020:8910692. doi: 10.34133/2020/8910692 (PMC7521026; doi:10.34133/2020/8910692)
Supplement: Supplementary 1 — Figure S1: deformation under 200 MPa of normal force observed by finite element analysis. Figure S2: analysis of changes in S and d under static (A) or sliding friction forces (B). Figure S3: changing process of the capacitance that the static converted to sliding friction force. Figure S4: the pulse-like signal under static friction force. (A) The pulse frequency responses in the 0 to 8.5 N range of static friction force. (B) The pulse shape through the custom-designed circuit. The pulse frequency decreased with the applied static friction force. Figure S5: comparison between traditional parallel structure and spiral structure. The spiral is centrosymmetric, which ensures the same sensitivity to shear force from any direction in plane. Table S1: the method for distinguishing shear forces. [file 8910692.f1.docx]

**Supplementary Materials**

**Highly selective biomimetic flexible** **tactile sensor**

**for neuroprosthetics**

Yue Li^1, 2^*, Zhiguang Cao^1^*, Tie Li^1, 2^^†^, Fuqin Sun^1^, Yuanyuan Bai^1^, Qifeng Lu^1^, Shuqi Wang^1^, Xianqing Yang^1^, Manzhao Hao^4^, Ning Lan^4^, Ting Zhang^1, 2,3†^

1: *i-Lab, Key Laboratory of multifunctional nanomaterials and smart systems, Suzhou Institute of Nano-Tech and Nano-Bionics (SINANO), Chinese Academy of Sciences (CAS), 38 Ruoshui Road, Suzhou, 215123, P. R. China.*

2: *School of Nano-Tech and Nano-Bionics, University of Science and Technology of China, 96 Jinzhai Road, Hefei, Anhui, 230026, P.R. China*

*3:Center for Excellence in Brain Science and Intelligence Technology,Chinese Academy of Sciences, Shanghai 200031, China*

4: *Laboratory of Neurorehabilitation Engineering, School of Biomedical Engineering and Institute of Medical Robotics, Shanghai Jiao Tong University, 1954 Huashan Road, Shanghai, 20030, P. R. China.*

** These authors contributed equally to this work: Yue Li and Zhiguang Cao.*

*† Corresponding author.Email:*[*tzhang2009@sinano.ac.cn*](mailto:tzhang2009@sinano.ac.cn) *(T. Zhang);* [*tli2014@sinano.ac.cn*](mailto:tli2014@sinano.ac.cn) *(T. Li).*

**Table of Contents:**

**Figure S1.** Deformation under 200 MPa of normal force observed by finite element analysis.

**Figure S2.** Analysis of changes in S and d under static (A) or sliding friction forces (B)

**Figure S3.** Changing process of the capacitance that the static converted to sliding friction force.

**Figure S4.** The pulse-like signal under static friction force. (A) The pulse frequency responses in the 0 to 8.5 N range of static friction force. (B) The pulse shape through the customed-designed circuit. The pulse frequency decreased with the applied static friction force.

**Figure S5.** Comparison between traditional parallel structure and spiral structure. The spiral is centrosymmetric, which ensures the similar sensitivity to shear force from any direction in plane.

**Table S1.** The method for distinguishing shear forces.

**Movie S1.** Weight perception of the flexible friction force sensors. A robotic hand was set to grasp a plastic bottle with a constant force. The capacitance of the sensor increased with the addition of water.

**Movie S2.** Slippage detection of the flexible friction force sensors. The flexible friction force sensor was mounted onto a wearable glove. The capacitance of the sensor decreased once slippage occurred.

**Movie S3.** Insensitivity to the normal force of the flexible friction force sensors. The capacitance of the sensor was constant under different standard weights placed on the sensor.

**Movie S4.** The bionic behavior of flexible friction force sensors. The flexible friction force sensor mounted onto a wearable glove. The glove was used to apply a sliding friction force. The output is the frequency output through the conversion circuits. The frequency increased once slippage occurred.

**Movie S5.** Changing of the capacitance as the static friction force converted to sliding friction force. A glove equipped with a Ruffini-ending-inspired sensor was put on hand to perform the action. The sensor was exerted static friction force at the beginning and then converted to slide mode.


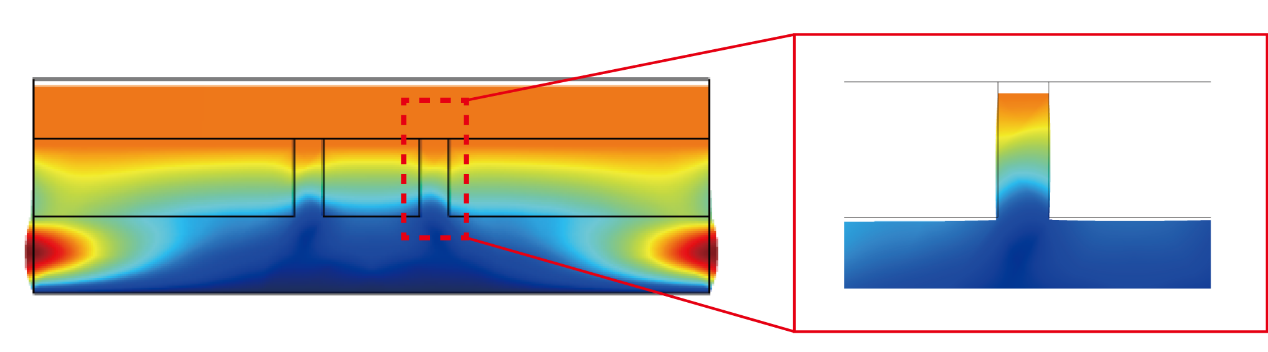


**Figure S1. Deformation under 200 MPa of normal force observed by finite element analysis.** Bulk structures made of PDMS and Ecoflex exhibit high resistance to external normal forces. The normal force added through the top silica glass and the bottom of the sensor was fixed. 200 MPa corresponds to the maximum normal force (20 N) in the experiment. The height of the electrodes decreased by only 6.75%, and the changes in d and l were too small to approximate to zero.

In the following equation:

$\Delta C=c-c_{0}=\frac{\varepsilon_{r}\varepsilon_{0}\left( h+\Delta h \right)\left( l+\Delta l \right)}{d+\Delta d}-\frac{\varepsilon_{r}\varepsilon_{0}hl}{d}$ (1)

h is the height of the electrodes, l is the length of the electrodes and d is the distance between two electrodes.

Because Δd and Δl were too small to approximate to 0 under 200 MPa, ΔC can be rewritten as

$\Delta C=\frac{\varepsilon_{r}\varepsilon_{0}\left( h+\Delta h \right)l}{d}-\frac{\varepsilon_{r}\varepsilon_{0}hl}{d}=\frac{\varepsilon_{r}\varepsilon_{0}\Delta hl}{d}$ (2)

Plugging Δh=-6.75% into equation (2),

$$\frac{\Delta C}{C_{0}}=\frac{\Delta h}{h}=-6.75\%$$

which is in accordance with the measured $\frac{\Delta C}{C_{0}}$ (-4.18%).


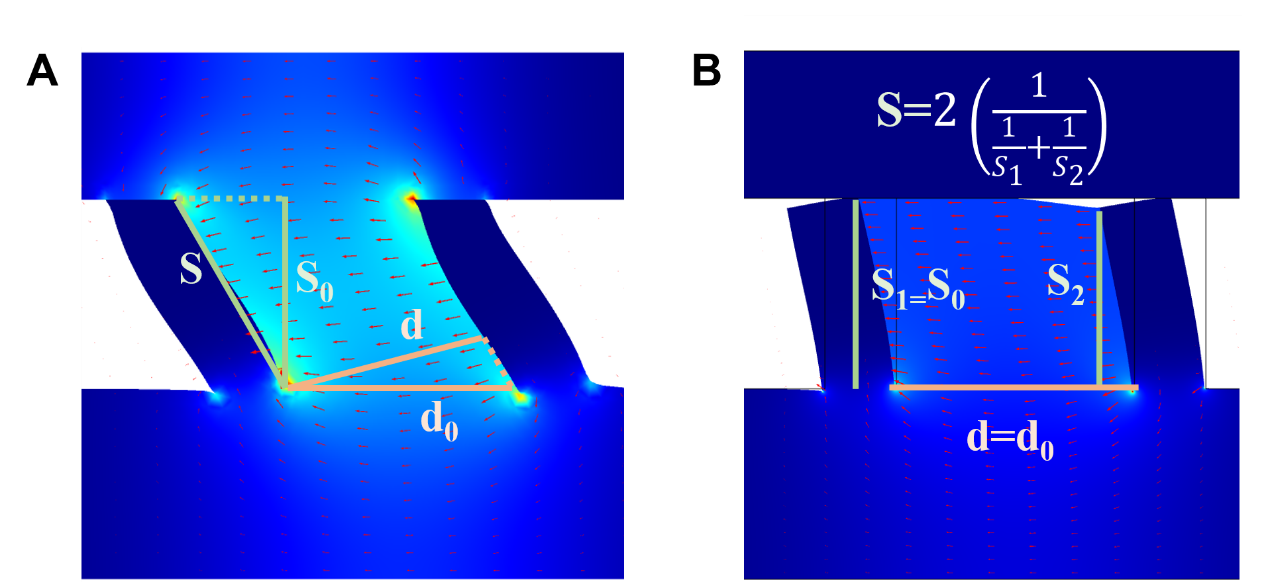


Figure S2. Analysis of changes in S and d under static (A) or sliding friction forces (B)

The equation of the plane-parallel capacitor, C$=\frac{\varepsilon_{r}\varepsilon_{0}S}{d}$, is derived from the definition of capacitance:

$C=\frac{Q}{U}$ (3)

C is the capacitance, Q is the charge held by the conductor and U is the voltage between two electrodes. To better understand the definition of S and d, the derivation process is described in detail.

According to Gauss’s Law

$\Phi_{E}=∯_{S} E*dS$ (4)

Where Φ_E_ is the electric flux through a closed surface S, E is the electric field, dS is a vector representing an infinitesimal element of area of the surface. More specifically, the infinitesimal area is regarded as planar and with area dS. The vector dS is normal to this area element and has magnitude dS. The above equation (4) can be modified to,

$E=\frac{Q}{\varepsilon S_{\perp}}$ (5)

Where $\varepsilon$ is permittivity and $S_{\perp}$ is the surface area that perpendicular to the electric field line.

So the voltage between two electrodes (U) can be expressed as

$U=Ed_{\parallel}=\frac{Qd_{\parallel}}{\varepsilon S_{\perp}}$ (6)

Where d is the distance that parallel to the electric field line.

Therefore,

$C=\frac{Q}{U}=\frac{\varepsilon S_{\perp}}{d_{\parallel}}$ (7)

Coupling of solid mechanics and electrostatic field was stimulated as supplementary materials. Electric field distribution after deformations are shown in Figure S2 separately.

For static friction force, electric field lines between two elongated electrodes were nearly perpendicular to electrodes. According to equation $C=\frac{Q}{U}=\frac{\varepsilon S_{\perp}}{d_{\parallel}}$ , compared with initial value,

d＜d_0_ S＞S_0_

So C＞C_0_ (Figure S2-A). That is, compared with the initial capacitance of sensor, capacitance will increase during loading static friction force.

Conversely, for sliding friction force, electric field lines between two curving electrodes remained horizontal, and the two polar plates have different surface areas perpendicular to the electric field lines, S_1_ and S_2_ respectively. And the charge density of each plate is

$\sigma=\frac{Q}{S}$ (8)

The electric field of the plate is

$E=\frac{\sigma}{\varepsilon}$ (9)

According to Gauss' law(using the “pillbox” surface), the electric field between the plates is determined by

$E\cdot S_{pillbox}=E\cdot2S_{circle}=\frac{1}{\varepsilon}\sigma S_{circle}$ (10)

One of the plate is

$E_{1}=\frac{\sigma_{1}}{2\varepsilon}=\frac{Q}{2S_{1}\varepsilon}$ (11)

The other plate is

$E_{2}=\frac{\sigma_{2}}{2\varepsilon}=\frac{Q}{2S_{2}\varepsilon}$ (12)

So the voltage between the plates is

$U=(E_{1}+E_{2})\cdot d=\frac{Q}{2\varepsilon}\frac{S_{1}+S_{2}}{S_{1}S_{2}}d$ (13)

According to equation (3)

$$C=\frac{Q}{U}=\frac{2\varepsilon S_{1}S_{2}}{d(S_{1}+S_{2})}$$

compared with initial value,

d=d_0_ $S=\frac{2S_{1}S_{2}}{S_{1}+S_{2}}=2\left( \frac{1}{\frac{1}{S_{1}}+\frac{1}{S_{2}}} \right)＜S_{0}=2\left( \frac{1}{\frac{1}{S_{1}}+\frac{1}{S_{1}}} \right)$ (S_0_=S_1_>S_2_)

So C＜C_0_. That is, compared with initial capacitance of sensor, capacitance will decrease under sliding mode.





Figure S3. Changing process of the capacitance that the static converted to sliding friction force.

According to the design principle of the Ruffini-ending-inspired sensors, the value of capacitance depends on the deformation of the spiral electrode microstructures, which are different under static or sliding friction forces. As shown in Figure 3B, if the sensor is exerted by sliding friction force, the capacitance will be smaller than the initial capacitance. On the contrary, the capacitance will be bigger than the initial capacitance if the sensor is applied with static friction force (Figure 3C). In theory, sliding occurs once the static friction force reaches to the threshold value and the deformation of microstructure changes simultaneously. Due to the rapid transition from maximum static friction to sliding friction, the corresponding capacitance decrease to the value that less than the initial capacitance instantly as soon as static friction change to sliding mode.

To verify the above assumption, a glove equipped with a Ruffini-ending-inspired sensor was put on hand to press the balance with sliding tendency and then perform the sliding action. The sensor was exerted by static friction force at the beginning and then converted to slide mode twice. As shown in Figure S3 and movie S5, capacitance dropped below the initial capacitance (C_0_) once sliding occurred, which proved the above assumption.


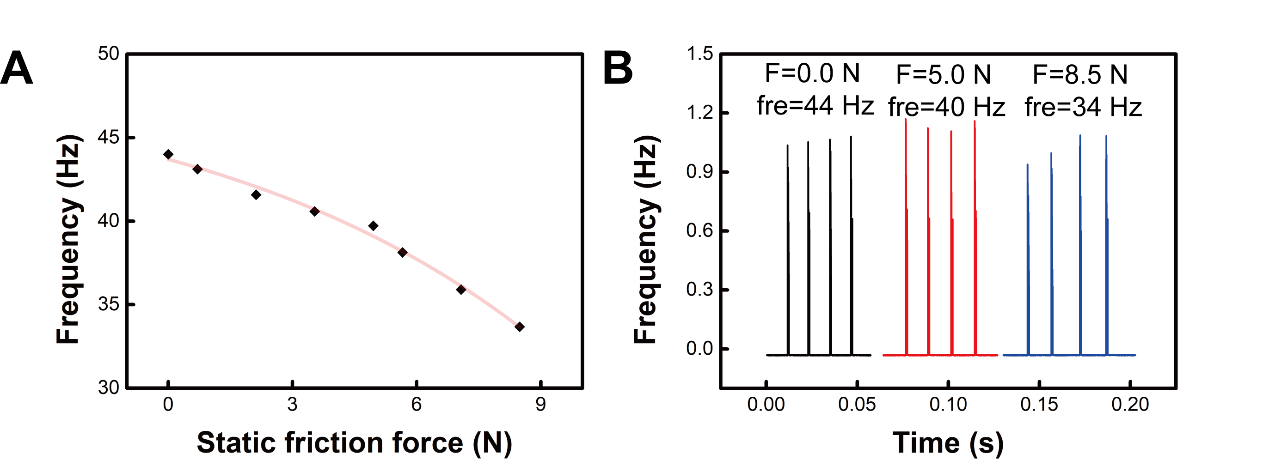


Figure S4. The pulse-like signal under static friction force. (A) The pulse frequency responses in the 0 to 8.5 N range of static friction force. (B) The pulse shape through the customed-designed circuit. The pulse frequency decreased with the applied static friction force.

Firstly, the designed circuit can also encode the static friction force based on its working principle. Pulse signals under static friction mode has also been investigated and included in the supplement materials. As shown in Figure S5, the spike frequency decreased as the capacitance increased due to the static friction force. The fitting formula of $f=-5.60*e^{\frac{x}{8.26}}+49.30$ was obtained with static friction loading in the range of 0 to ~8.5 N.

Secondly, the ability to discriminate static and sliding friction forces relies on the front-end flexible sensor. As introduced in the manuscript, the capacitance of the sensor increases with the static friction force but decreases under sliding mode. More specifically, compared with the initial capacitance of the sensor (C_0_), capacitance under sliding mode is less than C_0_. Conversely, capacitance under static friction force is larger than C_0_. Accordingly, we can distinguish the static friction force from sliding friction force based on the comparison with the C_0_.

Similarly, signals from A/D converter also can be discriminated by comparing with the initial frequency (f_0_). The circuit is designed to convert capacitance signal into physiological spike signals regardless of static friction or sliding friction forces, which follows the simplified formula (6):

$f=\frac{1}{a\sqrt{C}}$ (6)

where $a$ is a constant.

The initial capacitance (C_0_) under no shear force corresponds to the initial frequency (f_0_). According to the formula (6) and the relationship between capacitance and shear forces, the spike frequency increased as the sliding friction force increased. However, a decrease in spike frequency was observed when the static friction force increased. Therefore, if f＜f_0_, the shear force belongs to static friction force. Conversely, if f＞f_0_, the shear force is sliding friction force. The truth table for distinguishing shear forces is summarized in the following table S2.

Table S1. The method for distinguishing shear forces.

|  | Static friction force | Initial value | Static friction force |
| --- | --- | --- | --- |
| Capacitance | ＞C_0_ | C_0_ | ＜C_0_ |
| Frequency | ＜f_0_ | f_0_ | ＞f_0_ |


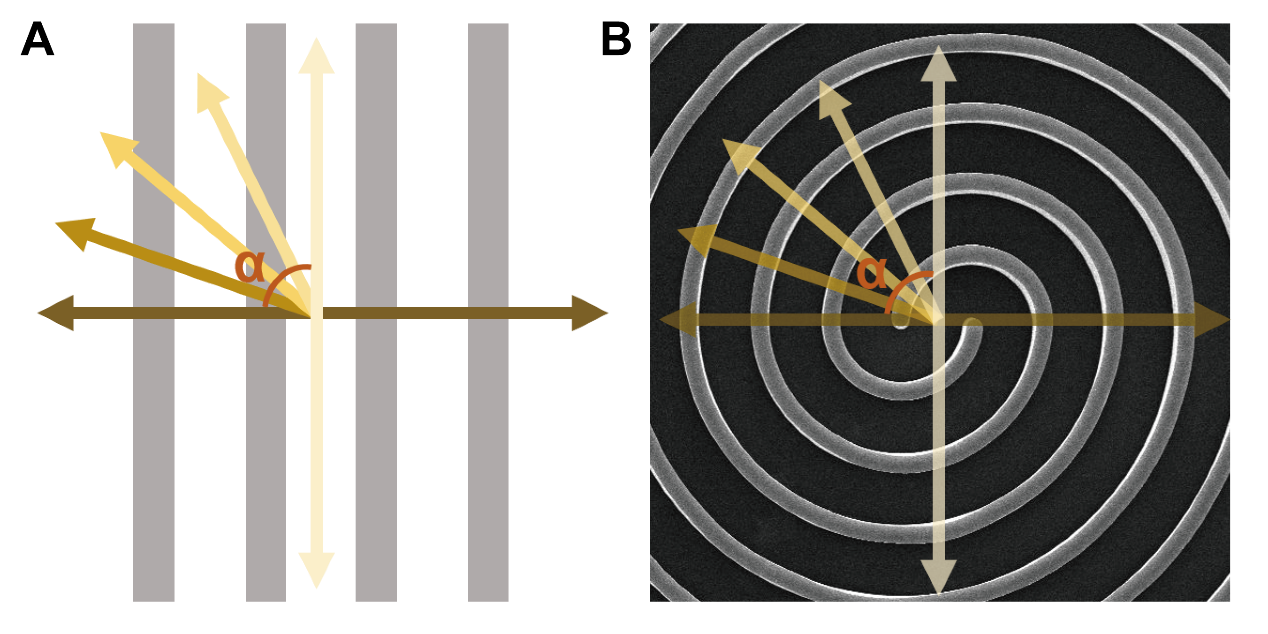


Figure S5. Comparison between traditional parallel structure and spiral structure. The spiral is centrosymmetric, which ensures the similar sensitivity to shear force from any direction in plane.

Human have the perception of multi-direction shear forces in plane. To imitate this ability, the capacitor sensor is designed as centrosymmetric structure. In detail, as shown in figure S6, the traditional linear type capacitor is axisymmetric, which is sensitive to the force perpendicular to electrodes in to plane (A direction) rather than parallel to the electrode (B direction). Besides, sensitivities of the sensor to shear forces from different directions within the α angle are different, which is difficult to judge the magnitude of force. However, the spiral is centrosymmetric, which ensures the similar sensitivity to shear force from any direction in plane.
